# An evaluation of the performance and suitability of R × C methods for ecological inference with known true values

**DOI:** 10.1007/s11135-017-0481-z

**Published:** 2017-02-03

**Authors:** Carolina Plescia, Lorenzo De Sio

**Affiliations:** 1 Department of Government, University of Vienna, Rathausstrasse 19/9, 1010 Vienna, Austria; 2 LUISS Guido Carli University, Viale Pola 12, 00198 Rome, Italy

**Keywords:** Ecological inference, Split-ticket voting, R × C contingency tables, Aggregate data

## Abstract

Ecological inference refers to the study of individuals using aggregate data and it is used in an impressive number of studies; it is well known, however, that the study of individuals using group data suffers from an ecological fallacy problem (Robinson in Am Sociol Rev 15:351–357, 1950). This paper evaluates the accuracy of two recent methods, the Rosen et al. (Stat Neerl 55:134–156, 2001) and the Greiner and Quinn (J R Stat Soc Ser A (Statistics in Society) 172:67–81, 2009) and the long-standing Goodman’s (Am Sociol Rev 18:663–664, 1953; Am J Sociol 64:610–625, 1959) method designed to estimate all cells of R × C tables simultaneously by employing exclusively aggregate data. To conduct these tests we leverage on extensive electoral data for which the *true* quantities of interest are known. In particular, we focus on examining the extent to which the confidence intervals provided by the three methods contain the true values. The paper also provides important guidelines regarding the appropriate contexts for employing these models.

## Introduction

Ecological inference can be defined as “the process of drawing conclusions about individual-level behavior from aggregate … data when no individual-level data are available” (Schuessler 1999: 10578). Ecological inference methods are relevant for all those applications where aggregate data are abundant, while individual-level data can be hard to collect. Given that in these situations aggregate data are readily available and can help researchers answer a multitude of theoretically interesting questions, the need arises to ascertain the accuracy and efficacy of the available methods to estimate disaggregated values starting from aggregate data. Many typical examples are related to voting behaviour, for which aggregate data are usually easy to collect: typical applications of ecological inference methods concern racial bloc voting, vote turnover tables, and split-ticket voting.

A typical formulation of an ecological inference problem is in terms of a cross-tabulation of two nominal variables (e.g. race and turnout) where marginals are known, but cell proportions are unknown (King 1997; Schuessler 1999). In the language of ecological inference analysis, 2 × 2 contingency tables represent binary data where the data are arrayed to create a table of two rows and two columns. While larger tables are usually referred to as R × C contingency tables. For all such classes of problems, ecological inference methods are able to estimate cell proportions at an aggregate (e.g. district) level, when marginals for analogous cross-tabulations are available for a number of units of analysis at a lower aggregation level (e.g. polling stations). Historically, the main methods for ecological inference have been Goodman’s (1953, 1959) ecological regression, King’s (1997) EI approach—originally developed for 2 × 2 tables, and later extended to the general R × C case (Rosen et al. 2001), and several other more recent techniques.

All such techniques have generated both great interest and a lively discussion, given their promise to produce reliable estimates based on information that is in principle plagued by the problem of *ecological fallacy*. While today 2 × 2 methods have been empirically evaluated (e.g., Wakefield 2004; Hudson et al. 2010), almost no empirical evaluations have characterized methods that face the issue of estimating larger tables. This is surprising given that the real world usually tends to present situations where data needs to be arrayed in tables with more than two columns and rows. Given their potential wide applicability, a test of their performance and suitability for ecological inference is necessary. In this paper, we contribute to this debate by performing a comparative test of three R × C ecological inference methods on a rather extraordinary dataset: a collection of electoral data (at the polling station level) for all districts in different countries, *where the true values of cell proportions are known.* In particular, we focus on estimation of cross-tabulations concerning the phenomenon of *split*-*ticket voting* (see below), in cases where *true values* are identified during the vote counting process, and then published by national electoral authorities. Our research strategy is straightforward: we assess the reliability of ecological inference methods by: (1) using them to estimate split-ticket voting matrices; (2) by comparing each estimated cell coefficient with the known true value. In particular, we test whether—and to what extent—true values fall within the 95% confidence intervals estimated by each method, with the expectation that this should happen in approximately 95% of the cases.

The rest of the paper is structured as follows. After this introductory section, we briefly contextualize the paper and discuss our research design. Then, we present our peculiar dataset. Section 4 presents the different estimation methods we compare. Section 5 outlines the main findings, and it is followed by a concluding section.

## The ecological fallacy problem

While not so frequently used in contemporary social science, ecological inference was understandably one of the fundamental tools of social science between the 19th and 20th centuries, before the development and diffusion of mass surveys (Achen and Shively 1995). In particular, techniques for ecological inference, such as *ecological correlation,* were popular in electoral research, one of the first fields of study where a wealth of aggregate data became widely available (e.g., Ogburn and Goltra 1919).

The end of this age of widespread use of ecological correlation came in the 1950s, with the identification by Robinson (1950) of the *ecological fallacy* problem. In short, Robinson showed that, at the aggregate level, the relationship between aggregate measures of individual-level variables—estimated through ecological regression—could even have the *opposite* sign as the true, individual-level relationship.^1^ Even if just a few years later Goodman identified conditions for avoiding ecological fallacy, developing a new model of *ecological regression* (1953, 1959), the importance of Robinson’s contribution, combined with the maturation of the mass survey as a powerful alternative to study individual-level attitudes and behaviour, led to a virtual “collapse of aggregate data analysis” (Achen and Shively 1995: 5). This led scholars “to avoid using aggregate data to address whole classes of important research questions” (King 1997: 5).

After several decades in which applications of ecological regression remained confined in few specialized sectors of electoral research—such as the estimation of vote turnover tables (see e.g., Corbetta et al. 1988), the publication of the book *A Solution to the Ecological Inference Problem* by Gary King which introduced a novel EI approach (King 1997) was received by great interest especially by political scientists. From then on, several other EI techniques and approaches were rediscovered, with the flourishing of numerous studies applied to different fields (King, Tanner and Rosen 2004). Nowadays, ecological inference methods are often used in applications related to voting behaviour, ranging from racial bloc voting, to vote turnover tables, and to split-ticket voting.

Yet, today, while contemporary 2 × 2 methods have been empirically evaluated (e.g., Wakefield 2004; Hudson et al. 2010), almost no empirical evaluations have characterized methods that face the issue of estimating larger tables. We contribute to this debate by comparing three R × C ecological inference methods on a dataset on split-ticket voting *where the true values of cell proportions are known*, hence, exploiting the comparison between aggregate-based estimates with individual-level true data that was the basis for Robinson’s seminal contribution. We will test, in particular, the reliability of estimation techniques, in terms of the extent to which estimated confidence intervals include the true values.

Our test is particularly relevant as, in multi-party systems, widespread, real-world applications related to split-ticket voting and vote turnover tables almost invariably require a R × C setup, i.e. estimating cell frequencies of contingency tables with multiple rows and columns. We are then extremely interested in model performance under these conditions. Are model estimates close to true values? Do true values lie in the estimated confidence intervals with the expected probability? Empirical answers to such questions will allow to assess the actual reliability of such ecological inference techniques when applied to real-world scenarios in multi-party systems. We present our data next.

## Data

In countries using mixed-member electoral systems voters usually cast two votes *simultaneously*, one for a national party under proportional rules (PR) and one for a local candidate under plurality rules, to elect the same legislative body. Voters are said to cast a *straight* ticket if they vote for the candidate of the same party for which they cast their PR vote; otherwise, they are said to cast a *split* ticket.^2^

In most cases, the two types of votes are counted and published separately, so that the percentages of straight and split ticket voting for each party-candidate pair cannot be directly assessed. However, there are cases where this is not true, and votes for parties and candidates are counted and published also in joint form. This effectively translates in the official publication, by electoral commissions, not only of the *marginals* concerning parties and candidates, but also of the *cell frequencies* of the party-candidate cross-tabulation. In particular, such data are routinely available since 2002—at the aggregate district level—for general elections in New Zealand, and became exceptionally available also for the 2007 election of the Scottish parliament.^3^ For our analysis, we collected electoral results from all polling stations in New Zealand for the elections in 2002, 2005 and 2008 and in Scotland for the 2007 elections. We then used these data to estimate coefficients of straight and split-ticket voting for each party at a higher “district” level, to be compared with official reports of split ticket voting available at the same level (*constituency* in Scotland, *electorate* in New Zealand). This extraordinary opportunity of knowing the true quantities of interest allows an empirical test where the estimates provided by ecological inference methods can be directly compared with the true values.

The main political parties in New Zealand that run for all the different elections considered in this paper and also ran candidates on the plurality tier of the ballot paper include on the left, the *Labour Party* and the *Greens* and on the right, the *National Party*, *New Zealand First* (NZF) and the *Association of Consumers and Taxpayers* (ACT); additionally there were many small parties contesting the elections that rarely also ran candidates. The political parties in 2007 in Scotland include the *Labour Party*, the *Scottish National Party* (SNP), the *Liberal Democrats* (Lib Dems) and the *Conservative Party*; beside these about six small parties stood for elections but almost never ran candidates for district seats.

Since ecological inference is essentially a problem of aggregation, comparing the performance of ecological inference methods across different types of contexts is highly important (Park et al. 2014). In this regard, the pooled dataset of the four aforementioned elections exhibits conspicuous variation (see Table 1). First, the size of the contingency R × C tables varies in each election, across districts as well as between countries. In general, the number of parties (i.e., No. of rows of our contingency tables) is constant across districts within each country but it varies across years of election. The number of candidates (i.e., No. of columns) instead varies significantly across districts and across elections: this provides a variation in contingency tables size, from a minimum of 10 × 4 in some Scottish districts to a maximum of 19 × 14 in some districts in New Zealand. For all estimation methods we first ran simulations for all parties as separate rows and for all candidates as separate columns (that we call *full forms*). We then ran a second set of simulations by collapsing rows and columns for parties and candidates obtaining less than 5% of the total vote at the district level (that we call *reduced forms*) and we investigate whether and how reducing the dimension of tables affects the results (The “Appendix” shows, for each election, which parties have been considered in the full form and which have been merged to get the reduced form).

**Table 1:** Summary of country, between-districts and within-district variation

Country	Year	No. of districts	No. of polling stations (range)	No. of parties (range)	No. of candidates (range)	Within-district average party variance (SDs) (range)
New Zealand	2002	69	25–645	14 (7–8)^a^	6–11 (3–7)^a^	10.33 (9.99)–542.81 (306.92)
	2005	69	24–691	19 (7–8)^a^	3–14 (3–8)^a^	13.55 (13.20)–599.14 (299.91)
	2008	70	27–681	19 (7–8)^a^	2–14 (3–7)^a^	11.45 (10.34)–417.39 (265.88)
Scotland	2007	73	22–103	16–25 (5–8)^a^	5–8 (5–8)^a^	15.48 (14.21)–226.57 (927.52)

^a^Numbers in parenthesis represent No. of rows or columns in reduced forms

Second, the number of subunits (here polling stations) used for the estimation has been shown to matter for the quality of the higher (district) level estimates: specifically the literature specifies a criterion of at least 2 subunits per coefficients (Corbetta et al. 1988; Corbetta and Parisi 1990; Biorcio and Natale 1991; Mannheimer 1993). While this criterion is often met for the estimation of 2 × 2 tables, it may not be satisfied for larger contingency tables and it is worth assessing whether the number of subunits affects the overall quality of the estimates. The number of polling stations in each district, in New Zealand ranges from 25 to 113 with only the seven Maori electorates, characterized by a much larger number of 645 polling stations in 2002, 691 in 2005 and 681 in 2008 election. For Scotland, the number of polling stations ranges from 22 to 103.

Another relevant source of variation is the within-district variance which refers to the fact that parties support varies considerably not only across districts but also across subunits within each district. We use a similar criterion as Park et al. (2014) and calculate the across-unit mean and variance of party support within each district with the expectation that a larger variance sets unfavourable conditions for the performance of ecological inference estimators.

In the empirical section we test the effect of all these sources of variation on the reliability of the estimates. The expectations are as follows:

- the smaller the contingency tables, the more reliable the estimates;
- larger ratios, calculated as the number of polling stations divided by the number of estimated coefficients, lead to more reliable estimates;
- the larger the across-unit variance, the less reliable the estimates.

## R × C methods

As previously anticipated, this paper tests three methods for ecological inference.^4^

### Ecological regression (Goodman 1953, 1959)

A long-standing method proposed to tackle the ecological fallacy issue is the Goodman’s method (Goodman 1953, 1959). Goodman formalizes the logic of the ecological inference in a simple regression model where the relationship to be studied is a linear one. Let *X* ^*i*^ be the proportion of the population in area *i* that belongs to group 1, 1 − *X* ^*i*^ the proportion of the population in area *i* that belongs to group 2, and *T* ^*i*^ the proportion of the population in area *i* with the characteristics or choice at issue. Goodman demonstrates that the accounting identity $T^{i} = \beta^{1i} X^{i} + \beta^{2i} (1 - X^{i} )$ holds exactly (see De Sio (2003) for an explanation of how the identity expands to larger tables). The key and most problematic assumption necessary for unbiasedness is that the parameters and *X* ^*i*^ are uncorrelated (King 1997; Tam Cho and Gaines 2004). Where this assumption does not hold the estimates will be biased, and even outside the deterministic bounds (e.g. that 105% of voters split their vote). Various remedies have been proposed to force the estimates to take only admissible values [see for instance Cleave et al. (1995)]. Given that in this paper we are mainly interested in testing whether or not the true values are inside the confidence intervals, the actual estimates are of less concern and no adjustment is being performed in the analysis below.

#### Applicability of assumptions

With reference to the specific problem at hand, the assumption of uncorrelation translates into a substantive assumption that, *at the polling station level*, the tendency to cast a split ticket vote among voters of one party (the cell coefficient) should not be correlated with the size of the party in the precinct. We see no reason in our data (and political context) why such assumption should be violated since the existing literature on split-ticket voting documents no relationship between split-ticket voting and the local strength of a political party at the polling station level (Karp et al. 2002; Burden 2009; Gschwend et al. 2003). In terms of the areal variations of cell probabilities, the presence of contextual variables may produce aggregation bias (Salway and Wakefield 2004). This is a particular problem for voting studies, as many potentially unmeasured variables, such as religion, age, can influence voting patterns. In our case, we have no specific expectations for the phenomenon of split-ticket voting to vary widely across ecological units; especially when—as in our case—estimates are obtained at the district level, which is still geographically small and of sufficient political homogeneity. As a result, we cannot identify any reason for major and systematic violations of the Goodman assumptions in our dataset.

### EI-MD method in its R × C formulation (Rosen et al. 2001)

Rosen and his co-authors propose two approaches for the estimation of R × C tables. The Bayesian approach extends the binomial-beta hierarchical model developed by King et al. (1999) from the 2 × 2 case to the R × C case. This model itself builds upon the seminal work of King (1997). In the first stage, the Rosen et al. (2001) method assumes that the stochastic component $T_{c}^{i}$ = ($T_{A}^{i}$, $T_{B}^{i}$, $T_{C}^{i}$) follows a Multinomial distribution with systematic component $\Theta = \sum\nolimits_{{}}^{r} {\beta_{rc}^{i} {\rm X}_{r}^{i} }$ where *r* = *A*, *B*, *C* and *c* = *A*, *B*, *C*. On the second level of this hierarchical model, the stochastic component $\beta_{rc}^{i}$ = $(\beta_{AA}^{i} ,\beta_{BA}^{i} , \ldots ,\beta_{rc}^{i} )$ follows a Dirichlet distribution with systematic component $\alpha_{rc}^{i} = \frac{{d_{r} \exp (\gamma_{rc} + \delta_{rc} Z_{i} )}}{{d_{r} (1 + \sum\nolimits_{j = 1}^{C - 1} {\exp (\gamma_{rj} + \delta_{rj} Z_{i} )} }} = \frac{{\exp (\gamma_{rc} + \delta_{rc} Z_{i} )}}{{1 + \sum\nolimits_{j = 1}^{C - 1} {\exp (\gamma_{rj} + \delta_{rj} Z_{i} )} }}$. In the third and final stage, the model assumes that the regression parameters (the $\gamma_{rc}^{i}$ and the $\delta_{rc}^{i}$) are a priori independent with a flat prior. The parameters $d_{r} ,r = 1, \ldots ,R,$ are assumed to follow exponential distributions with mean $1/\lambda$ (Rosen et al. 2001: 137–138). The marginals of the posterior distribution are obtained using the Gibbs sampler (Tanner 1996). As in the 2 × 2 case, the inferential procedure employs Markov chain Monte Carlo (MCMC) methods. As explained by Rosen et al. (2001) their approach can be computationally quite intense and for complex models the assessment of convergence may not be straightforward. They thus propose a simpler nonlinear least-squares approach (hereafter referred to as EI-MD) which is a direct approximation of their MCMC method but based on first moments rather than on the entire likelihood. As such, it provides quicker inference via nonlinear least-squares. This second approach is available in R software [either through the *Zelig* package (Wittenberg et al. 2007) or more recently the *eiPack* package (Lau et al. 2013)]. It should be noted that given that this strategy implements a frequentist approximation of the EI-MD Bayesian model, it is not Bayesian by design and does not require priors or starting values to be specified.

#### Applicability of assumptions

In general, the greater flexibility and robustness of this method—compared to ecological regression (King 1997; King et al. 2004; Rosen et al. 2001)—ensures that its assumptions should be met whenever the assumptions for ecological regression are met. As a result (see the discussion above) we do not assess in our data the risk of major violations of the assumptions for this method.

### EI-ML method (Greiner and Quinn 2009)

The third method we explore in this paper has been proposed by Greiner and Quinn (2009) (hereafter referred to as EI-ML). For each contingency table, the rows are assumed to follow mutually independent multinomials, conditional on separate probability vectors which are denoted by $\Theta_{r}$ for *r* = 1 to R (R being the number of rows in each contingency table). Each $\Theta_{r}$ then undergoes a multidimensional logistic transformation, using the last (right-most) column as the reference category.^5^ This results in R transformed vectors of length C; these transformed vectors are stacked to form a single $\omega$ vector corresponding to that contingency table. The omega vectors are assumed to follow (*i.i.d.*) a multivariate normal distribution (Greiner and Quinn 2009: 70–72). This method is structurally similar to the Rosen et al. (2001), although within-row relationships appear to be less constrained in the Greiner and Quinn (2009) as this model uses the stacked additive logistic normal distribution instead of mutually independent Dirichlet distributions.

As discussed by Greiner and Quinn (2009), seemingly innocuous differences to the prior distribution assumed for the model parameters can have large effects on the resulting posterior distribution and this on inference. Wakefield (2004) has demonstrated similar results for the 2 × 2 case. In this context, for the estimation of quantities of interest we use the default priors in the *R* × *CEcolInf* R package (Greiner et al. 2013) (that is a normal hyperprior distribution for the diagonal of the covariance matrix and Inverse-Wishart hyperprior for the diagonal of the matrix parameters) given that these seem to provide the closest possible values to the observed ones.

#### Applicability of assumptions

Here we offer similar considerations as those applicable to the Rosen et al. (2001) method above. Given that also this method offers a degree of flexibility and robustness that is superior to ecological regression (see Greiner and Quinn 2009), a result, even for this last method we do not identify reasons for major violations of the method’s assumptions.

## Findings

Each method reports the estimated means, standard deviation and the 95% confidence interval around the mean estimate. In our assessment below we focus in particular on how reliable the confidence intervals are. We then model the effect of all the aforementioned sources of variation on the reliability of the estimates. The idea is to assess whether the true levels of straight ticket voting is included in the 95% confidence interval provided by the three methods.

### The reliability of the confidence intervals

We start our foray into the results with an overall evaluations of the three methods.^6^ Table 2 reports the percentage of estimates inside the 95% CI by election and by party size. The confidence intervals of the EI-MD in its full form cover the true value only in about 30–40% of the cases; this percentage is generally higher in the reduced form. For the EI-ML this percentage is instead usually lower. Moving to the Goodman’s method, the confidence intervals covers the true value in about 30% of the case in the full form and slightly lower in its reduced form. While these values are consistent across election-year, Table 2 shows differences across party size. In particular we see that for the EI-MD method, the confidence intervals contain the true values more often for the smaller parties (Greens, NZF and to some extent ACT) than for the larger parties (Labour, National); and this seems to be true in both countries. This seems to be true also for the EI-ML and the Goodman methods where the difference between smaller and bigger parties is even more pronounced than for the EI-MD method. Moving to more specific sources of errors, Table 2 shows that the criteria of at least two polling stations per coefficient finds support in our data: the larger this ratio the more reliable the estimates except for the EI-ML method.

**Table 2:** Percentage observations inside 95% confidence intervals and RMSE, summary

	EI-MD full		EI-MD reduced		EI-ML reduced		Goodman full		Goodman red	
	% CI	RMSE	% CI	RMSE	% CI	RMSE	% CI	RMSE	% CI	RMSE
*By election*										
NZ 2002	39.8	0.157	40.9	0.157	29.9	0.238	28.8	0.227	25.9	0.226
NZ 2005	29.7	0.168	29.7	0.159	31.5	0.277	27.3	0.331	21.0	0.289
NZ 2008	32.3	0.147	35.5	0.137	26.4	0.263	29.8	0.285	23.6	0.286
STD 2007	34.5	0.143	49.1	0.131	11.7	0.163	23.4	0.222	26.1	0.243
*By party size*										
Large	30.9	0.126	40.6	0.121	17.2	0.171	17.9	0.187	13.5	0.195
Small	44.7	0.162	42.6	0.157	44.1	0.317	31.7	0.337	24.6	0.305
*Ratio*										
<1	32.8	0.156	28.9	0.167	31.8	0.289	17.7	0.313	11.5	0.277
1 < R < 2	42.6	0.184	41.5	0.139	25.3	0.226	12.8	0.232	19.0	0.249
>2	52.9	0.082	46.9	0.132	18.1	0.180	25.9	0.229	26.6	0.133

Recall that larger values of RMSE indicate less precise estimates. NZ stands for New Zealand. STD stands for Scotland. For the definition of large and small parties refer to footnote 3. R refers to the ratio calculated as the number of polling stations divided by the coefficients to be estimated. For the results of the EI-ML full refer to footnote 6

Table 2 also presents values of root mean square error (RMSE) which ranges from 0 to 1 with ‘0’ meaning that the estimated values are identical to the true values^7^; conversely, larger values of RMSE indicate less precise estimates. Generally speaking, Table 2 indicates that the models work best in estimating values for bigger parties when compared to smaller parties. Overall there is a striking result: on the one hand, the results for large parties are more precise in terms of RMSE evaluation. On the other hand however, the confidence intervals for the large parties are so narrow that they fail to include the true value in most of the cases.

In the most optimistic scenario, i.e., where the polling-station-per-estimated-coefficient ratio is above 2, the best performing method, i.e., EI-MD full, yields reliable estimates in only about 53% of the cases. Given that, on the grounds of model assumptions—and with no apparent major violation of model assumptions in our data, we should instead expect the estimated confidence interval to include true values roughly in 95% of the cases, these results cast serious doubts on the ability of such techniques to live up to their promises of accuracy. It must be said of course that our conditions are far from ideal. Most of the turnover tables we estimated are pretty large in size, leading to the necessity of estimating a large number of coefficients despite the limited number of polling stations; also, the lower variance for smaller parties reduces the amount of information that can be successfully exploited for the estimations. As a result, we deem worth investigating in more depth the *predictors* of unreliability. What are the conditions that increase the likelihood of obtaining reliable estimates?

### Predictors of unreliable confidence intervals

In this section we examine the conditions under which the estimated value lies outside the predicted bounds by focusing on the three main sources of variation discussed above: the size of the contingency table, the ratios and the across-unit variance. Specifically, we run logit models in which the dependent variable takes a value of 0 every time the true value lies outside the confidence interval and 1 otherwise.

The results in Table 3 indicate that the estimated 95% confidence interval is less likely to contain the true values in the case of larger contingency tables both in terms of number of columns and rows, however, the results are only statistically significant for the reduced forms of the models and in the case of rows but not for columns. On the opposite the number of polling stations is positively correlated with precise confidence intervals. The variance across polling stations within each district is negatively associated with precise confidence intervals despite the fact that the coefficient for this variable is statistically significant only in the case of the EI-MD method. The second set of models takes into account the ratios obtained using three of the independent variables in the first set of models: the number of polling stations divided by the number of coefficients to be estimated (number of columns multiplied by the number of rows). The results in Table 3 indicates that the ratio is overall positively related to the reliability of the estimates for all methods except the EI-ML. There are also two important differences across models worth to be discussed. First, while the variance is much more important to explain the error for the EI-MD model compared to the others models, the ratio criterion is way more important for the Goodman method. Also, using the robustness statistics at the bottom of Table 3, it is clear that the features we take into account explain much more of the variability of the Goodman method when compared to the other two methods.

**Table 3:** Predictors of reliable confidence intervals (logit regression)

	EI-MD full		EI-MD reduced		EI-ML reduced		Goodman full		Goodman red	
	Model 1	Model 2	Model 3	Model 4	Model 5	Model 6	Model 7	Model 8	Model 9	Model 10
No. of columns	−0.000 (0.039)		−0.016 (0.080)		0.055 (0.074)		−0.062 (0.034)		−0.048 (0.105)	
No. of rows	−0.020 (0.021)		−0.276* (0.112)		−0.448*** (0.121)		−0.042 (0.022)		−0.324* (0.153)	
No. polling stations	0.003* (0.001)		0.004* (0.002)		0.001 (0.001)		0.010* (0.004)		0.012* (0.005)	
Variance	−0.617 (2.352)	−1.124*** (0.251)	−3.704* (1.854)	−5.309** (1.787)	−0.138 (2.021)	−2.662 (2.030)	−0.487 (1.618)	−0.991 (1.588)	−1.157 (1.858)	−1.932 (1.847)
Ratio		0.180** (0.064)		0.104*** (0.023)		0.046 (0.042)		1.297** (0.433)		0.384* (0.191)
Constant	−0.454 (0.313)	−0.641** (0.215)	1.639* (0.671)	−0.140 (0.192)	−4.494*** (0.741)	−1.254*** (0.240)	−1.654** (0.531)	−0.606* (0.275)	−2.875*** (0.838)	−1.087** (0.368)
*N*	1302	1302	1302	1302	1177	1177	1260	1260	1260	1260
Nagelkerke	0.14	0.09	0.39	0.24	0.26	0.04	0.54	0.45	0.40	0.32
AIC	1671.448	1671.952	1701.950	1712.235	1333.634	1347.466	1382.122	1386.215	1216.894	1218.872
LL	−830.724	−832.976	−845.975	−853.117	−661.817	−670.733	−686.061	−690.107	−603.447	−606.436

Standard errors, clustered by district, in parentheses: * *p* < 0.05; ** *p* < 0.01; *** *p* < 0.001. Adding fixed effect by country leads to a slight increase in the predicted power of the models but do not change substantive conclusions

## Discussion and conclusion

Electoral behavior research is not unique in that researchers often need to use aggregate data to infer individual-level relationships. This is either because surveys are not available or because the main interest lies in the geographical variation of specific patterns for which surveys are of no avail. Because aggregate data are readily available and can help researchers answer a multitude of theoretically interesting questions, the need arises to ascertain the accuracy and efficacy of the available methods to estimate disaggregated values starting from aggregate data.

As of today, there has been little research on the accuracy of methods which extend ecological inference to situations where data need to be arrayed in tables with more than two rows and columns. Benefitting from the rich data available for New Zealand and Scotland, this paper has empirically evaluated the performance and suitability of the Rosen et al. (2001) and the Greiner and Quinn (2009) models for ecological inference and R × C tables and additionally compare these with the long-standing Goodman’s method.

From the analysis conducted in this paper, a number of observations are noteworthy. First, using RMSE we find that the EI-MD model perform relatively better than the other two methods when comparing estimates of the quantities of interest with the true values. Yet, values of RMSE are in most other cases quite large considering that they relate to quantities that are in the 0–1 range. It has been noted, in this regard, that the lower the amount of information available during the estimation process, the less precise the estimations will be: estimates for small parties are thus consistently less precise than those for bigger parties. For this reason, a linear error parameterization or conditioning the estimates on the EI standard errors may prove a useful strategy. These adjustments are particularly relevant in the context of second-stage regression analysis, when the researcher’s aim is to use the point estimates as dependent variable in regression models to investigate for instance the variation of straight-ticket voting across districts (Herron and Shotts 2003; Adolph et al. 2003).

Second, in most of the cases, the confidence intervals as provided by the three methods fail to include the true values. More specifically, with regard to the sources of error we analysed we found that: (a) the smaller contingency tables, the more reliable the estimates; (b) larger ratios, calculated as the number of polling stations divided by the number of estimated coefficients, lead to more reliable estimates; and (c) the larger the variance, the less reliable the estimates. Albeit differences exist across the three methods in the extent to which these sources of error effect the results. Hence, one fruitful extension of this study concerns the possibility of correcting the reliability of the confidence intervals and this is true for all methods investigated in this paper. Another extension is to attempt reducing the amount of estimation time needed to obtain values of interest, a problematic issue when applying the Rosen et al. (2001) and the Greiner and Quinn (2009) method. Attempts to parallelize sequential loops by debugging the R code in the provided packages have not produced reassuring results so far.

To sum up, our findings indicate that caution is warranted when using ecological inference methods. This is especially true in those cases where the estimations involve large contingency tables, and/or the polling station-coefficient ratio is small and very small parties are present because our study shows that in these cases especially estimates will be biased and the estimated confidence intervals not reliable as declared.

## Appendix

Below we present the list of parties considered in the full forms. The parties in italics are those merged to obtain the reduced forms. In the reduced forms we exclude the parties that have obtained less than 5% of the total vote at the district level. Given that the decision to exclude the parties below the 5% threshold is done at the district level, some of the parties in italics may have been included in one or two districts per year of election.

### New Zealand 2002

Labour Party (Lab), National Party (Nat), New Zealand First Party (NZF), ACT New Zealand (ACT), Green Party (GP), United Future (UF), *Jim Anderton’s Progressive Coalition Progressive), Christian Heritage Party (ChrHeritage), Outdoor Recreation NZ (Outdoor), Alliance, Aotearoa Legalise Cannabis Party (Legalise), Mana Maori Movement (Mana), OneNZ Party, NMP.*

### New Zealand 2005

Lab, Nat, NZF, GP, *Maori, UF, ACT, Progressive, Destiny New Zealand, Legalise, ChHeritage, Alliance, New Zealand Family Rights Protection Party (Family), Democrats for Social Credit (Social), Libertarianz, Direct Democracy Party, 99 MP Party, OneNZ Party, The Republic of New Zealand.*

### New Zealand 2008

Nat, Lab, GP, ACT, *Maori, Progressive, UF, NZF, The Bill and Ben Party, Kiwi, Legalise, New Zealand Pacific Party (Pacific), Family Party, Alliance, Social, Libertarianz, Workers Party, RAM, The Republic of New Zealand Party.*

### Scotland 2007

Scottish National Party (SNP), Labour, Conservative, Liberal Democrats (Lib Dems), *Scottish Green, Scottish Senior Citizens, Solidarity, Scottish Christian, BNP, Christian People, Socialist Labour, Scottish Labour, Scottish Socialist, UKIP, Publican Party, Scottish Unionist, Scottish Voice, Save Our NHS Group, Free Scotland, Had Enough Party, Scottish Enterprise, Scottish Jacobite Party, SACL, Communist, Independent Green Voice, Socialist Party.*

## Footnotes

1 One of the two key examples provided by Robinson concerned the relationship between *foreign birth* and *illiteracy.* At the individual level (observable in its true values, thanks to census data) the relationship was *positive,* i.e., immigrants were more illiterate than native-born, in line with theoretical expectations. However, at the aggregate level (both when aggregating by state or by larger geographical divisions) the relationship was *negative*, i.e. states with more immigrants had *lower* levels of illiteracy. This paradox—easily explained by the tendency of immigrants to concentrate in areas with higher economic development and thus higher literacy—clearly demonstrated the problem of *ecological fallacy*, i.e. the *aggregation bias* (King 1997) that emerges when we infer relationships at the individual level based on aggregate data (Robinson 1950, 354).

2 Voting solely for either a party or a candidate is possible. As discussed by Benoit et al. (2004) the observed totals of valid votes for different ballots always differ slightly, mainly because of different rates of invalid ballots. As for Benoit et al. (2004) we took the total number of votes to be the midpoint between the two ballot totals in the rare occasions when this was a problem.

3 Sources: Electoral Commission, New Zealand (www.electionresults.org.nz); last accessed July 2015. Scotland Electoral Office, Scotland, data available upon request from the authors.

4 Several other methods have been proposed for the estimation of R × C tables [see for instance King et al. (2004), Park et al. (2014), Elff et al. (2008), Forcina et al. (2012), Colombi and Forcina (2016)]. Our exclusive focus on the Goodman (1953), Rosen et al. (2001) and the Greiner and Quinn (2009) methods is due to several reasons. First, the three methods we examine in this paper rely exclusively on aggregate-level data; on the contrary, other methods require also individual-level data that in several instances are not available. Second, the methods tested here are readily available using R packages. As a result, a test of their performance will benefit a large number of potential users. Third, all three methods allow a series of important extensions, e.g. use of covariates, not usually available for other methods. Conditioning values of interest on covariates to ‘control’ for patterns of systematic variation at the unit of observations may be particularly important when voting is susceptible to aggregation bias like racial voting (Voss 2004). In our specific case, we do not use covariates because our unit is the polling station, i.e., subunits of cities or towns, and finding covariates at this level means finding reasons why values of straight-ticket voting are systematically different across streets of the same town which is undoubtedly a challenging task. In addition, split-ticket voting as discussed among others in Burden (2009) and Plescia (2016) is not as sensitive to the choice of the covariates as other electoral phenomena.

5 In their article Greiner and Quinn (2009: 70) choose the ‘abstain’ column as reference category. In the results presented below we use as reference a residual category representing the sum of party vote cast for parties receiving less than 2% of the vote but it is important to stress that changing this reference category did not alter the results significantly.

6 The EI-ML method results without collapsing columns are not shown in Table 2 as it was not feasible to estimate quantities of interest for all districts in a specific year of election. In other words, the EI-ML usually fails to reach convergence when the estimation tables have a number of rows and/or columns that exceeds 10. Nevertheless, the quantities in those few cases where the estimates were obtained did not show major variations before and after collapsing columns, as it is the case for the EI-MD model.

7 RMSE measures the differences between the estimated and the true observed values and it enables us to assess both the accuracy of the point estimates (i.e. how biased the estimator is) and the efficiency of point estimators. It is based on the formula $R - MSE = \sqrt {mean(\beta_{{}}^{estimated} - \beta_{{}}^{true} )^{2} }$ (see also Liu 2007 foe a similar approach).
